# GDBIG: A Pioneering Birth Cohort Genomic Platform Facilitating Intergenerational Genetic Research

**DOI:** 10.1093/gpbjnl/qzaf045

**Published:** 2025-05-15

**Authors:** Shujia Huang, Chengrui Wang, Mingxi Huang, Jinhua Lu, Jian-Rong He, Shanshan Lin, Siyang Liu, Huimin Xia, Xiu Qiu

**Affiliations:** Division of Birth Cohort Study, Guangzhou Women and Children’s Medical Center, Guangzhou Medical University, Guangzhou 510623, China; Division of Birth Cohort Study, Guangzhou Women and Children’s Medical Center, Guangzhou Medical University, Guangzhou 510623, China; Division of Birth Cohort Study, Guangzhou Women and Children’s Medical Center, Guangzhou Medical University, Guangzhou 510623, China; Division of Birth Cohort Study, Guangzhou Women and Children’s Medical Center, Guangzhou Medical University, Guangzhou 510623, China; Provincial Clinical Research Center for Child Health, Guangzhou 510623, China; Division of Birth Cohort Study, Guangzhou Women and Children’s Medical Center, Guangzhou Medical University, Guangzhou 510623, China; Provincial Clinical Research Center for Child Health, Guangzhou 510623, China; Department of Obstetrics and Gynecology, Guangzhou Women and Children’s Medical Center, Guangzhou Medical University, Guangzhou 510623, China; Division of Birth Cohort Study, Guangzhou Women and Children’s Medical Center, Guangzhou Medical University, Guangzhou 510623, China; Department of Women’s Health, Provincial Key Clinical Specialty of Woman and Child Health, Guangzhou Women and Children’s Medical Center, Guangzhou Medical University, Guangzhou 510623, China; School of Public Health (Shenzhen), Shenzhen Campus of Sun Yat-sen University, Shenzhen 518107, China; Provincial Clinical Research Center for Child Health, Guangzhou 510623, China; Guangdong Provincial Key Laboratory of Research in Structural Birth Defect Disease, Guangzhou Women and Children’s Medical Center, Guangzhou Medical University, Guangzhou 510623, China; Department of Pediatric Surgery, Guangzhou Women and Children’s Medical Center, Guangzhou Medical University, Guangzhou 510623, China; Division of Birth Cohort Study, Guangzhou Women and Children’s Medical Center, Guangzhou Medical University, Guangzhou 510623, China; Provincial Clinical Research Center for Child Health, Guangzhou 510623, China; Department of Women’s Health, Provincial Key Clinical Specialty of Woman and Child Health, Guangzhou Women and Children’s Medical Center, Guangzhou Medical University, Guangzhou 510623, China

**Keywords:** BIGCS, Genomic database, Genetic variant, Genotype imputation, Genome-wide association study

## Abstract

High-quality genome databases derived from large-scale, family-based birth cohorts are vital resources for investigating the genetic determinants of early-life traits and the impact of early-life environments on the health of both parents and offspring. Here, we established a genomic platform for the Born in Guangzhou Cohort Study (BIGCS), the Genome Database of BIGCS (GDBIG), which represents the first birth cohort-based genomic database in China and is designed to facilitate intergenerational genetic research. Based on the phase I results of the BIGCS, GDBIG includes low-coverage (∼ 6.63×) whole-genome sequencing (WGS) data and extensive pregnancy phenotypes from 4053 Chinese participants. These participants are from 30 of China’s 34 provincial-level administrative divisions, encompassing Han and 12 minority ethnic groups. Currently, GDBIG provides a range of services, including allele frequency queries for 56.23 million variants across two generations, a genotype imputation server featuring a high-quality family-based reference panel, and a genome-wide association study (GWAS) meta-analysis interface for various maternal and infant phenotypes. The GDBIG database addresses the lack of Asian birth cohort-based genomic resources and provides a valuable platform for conducting genetic analysis, accessible online or via application programming interfaces at http://gdbig.bigcs.com.cn/.

## Graphical abstract

**Figure:**
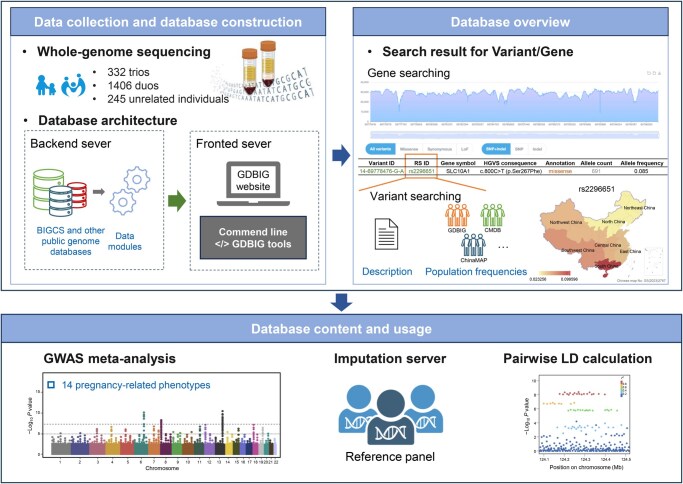

## Introduction

Genetic variation plays a crucial role in determining individual susceptibility to most human diseases [1]. Understanding the relationship between sequence variation and disease predisposition is therefore a powerful strategy for uncovering the fundamental processes involved in disease pathogenesis, treatment, and prevention. Recent advancements in sequencing technologies [2,3], analytical methodologies [4–7], and large-scale international and national genome sequencing initiatives [8–12] have greatly facilitated the identification of causal genes and variants. However, most genome sequencing projects to date have focused on unrelated adult individuals [8–12]. These efforts have contributed valuable online resources, such as Trans-Omics for Precision Medicine (TOPMed) [11,13], China Metabolic Analytics Project (ChinaMAP) [14], NyuWa Genome resource [15], and Stroke Omics Atlas (STROMICS) [12], which provide platforms for variant querying and genome imputation. While these platforms have substantially advanced genetic research, their focus on unrelated individuals has limited our understanding of the genetic determinants of traits that manifest early in life and the impact of early-life exposures on both short-term and long-term health outcomes for parents and offspring. To bridge these gaps, genome sequencing studies based on birth cohorts that recruit families in trios or duos, with longitudinal follow-up, are essential [16,17].

In recent years, several international and regional birth cohort studies have been initiated to explore the complex interplay between genetic factors and early-life environmental influences on lifelong health trajectories. These projects include those using array genotyping techniques to assay participants, such as the Early Growth Genetics (EGG) Consortium [18], the Avon Longitudinal Study of Parents and Children (ALSPAC) [19], the Hyperglycemia and Adverse Pregnancy Outcome (HAPO) study [20] involving multi-ethnic populations, the Olmsted County Birth Cohort (OCBC) [21], the Norwegian Mother and Child Cohort Study (MoBa) [22], and the Danish National Birth Cohort (DNBC) [23,24]. Additionally, several studies, such as the Born in Guangzhou Cohort Study (BIGCS) [25] and the China National Birth Cohort (CNBC) [26], have employed whole-genome sequencing (WGS) strategies.

A comprehensive genomic database that reports data from birth cohort studies and provides tools for their genetic analysis would be highly valuable. Such a database would not only offer genetic and genomic information similar to that available from genomic studies of unrelated individuals [8–12], but also enable intergenerational research that is not possible in studies of unrelated individuals, especially for underrepresented populations such as Asians. Despite the availability of various birth cohort studies, a comprehensive genetic database has yet to be established.

In this study, we present the Genome Database of BIGCS (GDBIG), a pioneering birth cohort-based genomic database and platform, developed using data from BIGCS, the largest birth cohort study in Asia. BIGCS was initiated in 2012 in Guangzhou, China, and has since recruited over 60,000 duo and trio families, with deep phenotypic data collected starting with pregnant women at less than 20 weeks of gestation and continuing with their children through age 18 [25]. GDBIG aims to provide user-friendly access to genomic, genetic, and phenotypic analytical data, along with genomic utilities based on genome sequencing and phenotypic data from BIGCS. The current GDBIG database includes phase I data from BIGCS, encompassing 4053 healthy individuals in duos and trios (332 trios, 1406 duos, and 245 unrelated individuals), all sequenced using WGS. The database currently includes information on 56,230,613 biallelic variants, comprising 51,052,456 single nucleotide polymorphisms (SNPs) and 5,178,157 insertions/deletions (Indels).

The primary functions of GDBIG are as follows. First, it provides a query interface allowing users to search for overall and provincial-level allele frequencies of genetic variants detected through WGS of BIGCS participants, along with visualization tools and genomic application programming interface (API) query functionalities. Second, it offers a genotype imputation server based on a high-quality reference panel, ensuring more accurate genotype imputation for individuals of similar ancestry, facilitated by the involvement of family members. This allows for precise long-range phasing. Lastly, it provides a genome-wide association study (GWAS) meta-analysis interface for numerous maternal and infant phenotypes cataloged from GWAS analyses conducted in BIGCS. Users can access and analyze the data online through the website (http://gdbig.bigcs.com.cn/) or via the genomic API (https://github.com/BIGCS-Lab/GDBIGtools).

## Data collection and database construction

### Data collection and processing

The GDBIG was established using data from the BIGCS, which included 4053 healthy participants: 332 trios (comprising fathers, mothers, and offspring), 1406 duos (14 father–offspring and 1392 mother–offspring pairs), and 245 unrelated individuals (70 children, 150 adult females, and 25 adult males). All participants were recruited through the BIGCS program at the Guangzhou Women and Children’s Medical Center (GWCMC). The parents ranged in age from 20 to 45 years, with 83.7% being mothers and 16.3% fathers. Among the children, 53.6% were male and 46.4% were female. The cohort was ethnically diverse, with 98% Han Chinese and participants from 12 minority ethnic groups. Participants were from 30 of China’s 34 provincial-level administrative divisions. Cantonese speakers represented the largest linguistic group, followed by Mandarin, Hakka, and Min speakers (Figure S1; Table S1).

Whole blood samples were collected from adults, and cord blood samples were collected from infants, with low-coverage WGS performed on these samples, achieving an average sequencing depth of approximately 6.63×. Data preprocessing involved removing poor-quality reads, followed by alignment and variant calling using the Genome Analysis Toolkit (GATK) best practice joint calling protocol. After variant quality score recalibration (VQSR), stringent quality control filters were applied. Multi-allelic variants were excluded, and additional filtering was performed to remove SNPs located in the low-complexity regions of GRCh38. The final set included 56,230,613 biallelic variants, comprising 51,052,456 SNPs and 5,178,157 Indels.

Variant distribution by allele frequency was as follows: 22,276,474 (39.62%) were singletons [allele count (AC) = 1], 11,919,262 (21.20%) were doubletons (AC = 2), 9,648,220 (17.16%) were very rare variants [AC > 2 and allele frequency (AF) ≤ 0.1%], 5,279,786 (9.39%) were rare variants (0.1% < AF ≤ 1%), 1,907,131 (3.39%) were low-frequency variants (1% < AF ≤ 5%), and 5,199,740 (9.25%) were common variants (AF > 5%). Approximately 32.56% of the variants [18,308,033 SNPs with the transition-to-transversion (Ts/Tv) ratio of 1.46] were not reported in the Single Nucleotide Polymorphism Database (dbSNP, build 154) maintained by the National Center for Biotechnology Information (NCBI). Moreover, 93.4% of the variants were classified as singletons or doubletons (AC ≤ 2). These variants were further annotated using Variant Effect Predictor (VEP) with default parameters [27]. More detailed descriptions of the variant and data quality control processes can be found in our associated published paper [28].

### Database architecture and web server implementation

**Figure 1:**
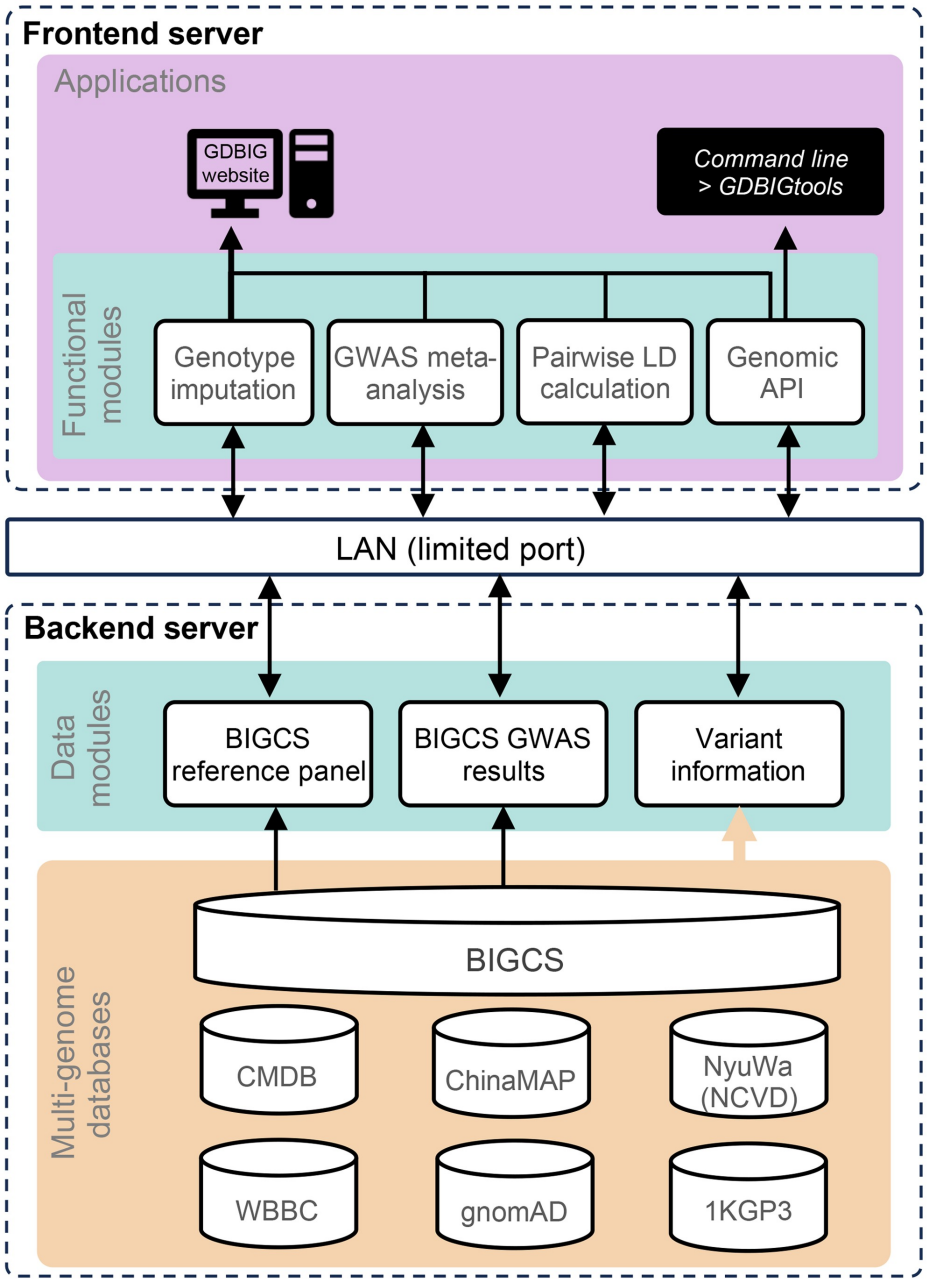
**Architecture of the GDBIG website and database **The GDBIG website is divided into two components: the frontend server and the backend server. Genomics data are securely stored in the backend server and cannot be accessed directly from the frontend. Data transfer is restricted to a controlled permission port within the LAN. GDBIG comprises four functional modules, providing users with online access and a genomic API for variant searching, genotype imputation, GWAS meta-analysis, and pairwise LD calculation via the frontend server interface. The backend server stores variant information from BIGCS, the BIGCS reference panel, and BIGCS GWAS results. Additionally, six other human genome variation databases (CMDB, ChinaMAP, NyuWa, WBBC, gnomAD, and 1KGP3) are integrated into the backend, enabling allele frequency comparison with the BIGCS dataset. GWAS, genome-wide association study; GDBIG, the Genome Database of BIGCS; BIGCS, the Born in Guangzhou Cohort Study; LAN, local area network; API, application programming interface; LD, linkage disequilibrium; CMDB, Chinese Millionome Database; ChinaMAP, China Metabolic Analytics Project; WBBC, Westlake BioBank for Chinese; gnomAD, Genome Aggregation Database; 1KGP3, 1000 Genomes Project Phase 3; NCVD, NyuWa Chinese population variant database.

Based on the genomic data from the BIGCS, we employed the following information technology (IT) strategies to construct GDBIG. Data security was ensured through the implementation of a deployment scheme consisting of two separate servers: one for the frontend application and the other for the backend. This configuration restricted the transfer of genomic data exclusively through a controlled permission port within the local area network (LAN) between these two servers (**Figure 1**).

For the backend, MongoDB (https://www.mongodb.com/) was selected as the genome database engine. MongoDB is a widely used, open-source, document-oriented non-relational database specifically designed for storing large-scale datasets. This choice allows for secure authentication and efficient management of data access, updates, and replenishment. Furthermore, GDBIG integrates variant data from multiple existing databases, including Chinese Millionome Database (CMDB) [29], ChinaMAP [30], NyuWa Genome resource [15], Westlake BioBank for Chinese (WBBC) [30], Genome Aggregation Database (gnomAD) [31], and 1000 Genomes Project Phase 3 (1KGP3) [8]. This integration provides a valuable resource for cross-population comparisons of variation spectra and supports researchers in their analyses.

The frontend interface of GDBIG utilizes Gin as the web framework (https://gin-gonic.com/) and My Structured Query Language (MySQL) for managing user information and permissions (https://www.mysql.com/). Gin is a high-performance, open-source Hypertext Transfer Protocol (HTTP) web framework written in Golang (Go, https://go.dev/), while MySQL is the most widely used free relational database management system globally, making it well-suited for managing user registration and access control. To visualize the data within GDBIG, the platform uses ECharts (https://echarts.apache.org/), a JavaScript library and open-source, cross-platform toolkit for data visualization. Due to legal protections of human genetic data in China, researchers are required to provide basic registration and login information before gaining access to the retrieval and analysis modules of the database.

## Database content and usage

### Searching and browsing variants on the website

The GDBIG browser provides access to detailed variant information sourced from the GDBIG database, including chromosomal positions, reference alleles, mutated alleles, biological consequences, overall mutated allele frequencies, and regional-level and provincial-level allele frequencies across seven geographical areas in China: North, Northeast, Northwest, East, Central, Southwest, and South. Users can access this information through the search page on the GDBIG website (http://gdbig.bigcs.com.cn/) by querying gene symbols, transcript identification (ID), reference SNP ID (RS ID) in dbSNP of variants, genomic regions, or chromosomal positions. The website ensures fast retrieval of variation information, which is presented with clear visualizations for ease of understanding.

**Figure 2:**
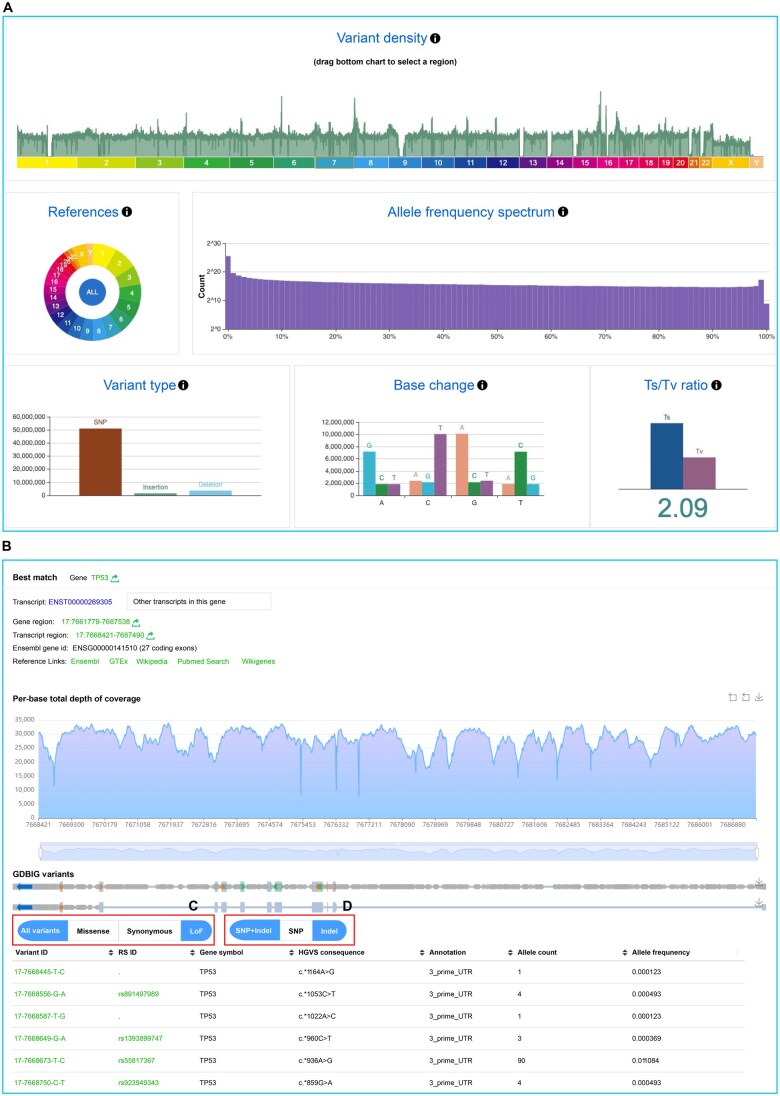
**Distribution of variants and example of gene search results in GDBIG** **A**. Overview page displaying summary statistics of genomic variants in GDBIG. **B**. Screenshot of a gene search for *TP53*, with the resulting variants within the *TP53* gene listed below. **C**. and **D**. Users can click on buttons to select specific functional annotations (C) or to choose different types of variants (SNPs or Indels) (D). SNP, single nucleotide polymorphism; Indel, insertion/deletion; LoF, loss of function; Ts, transition; Tv, transversion.

On the overview page, a dynamic display provides summary data on BIGCS variants, allowing users to select specific chromosomes or zoom in on particular regions to access detailed variation information quickly (**Figure 2**A). When searching by genomic region or gene ID, the results include summary information, coverage read depth for each position, transcript location distribution (if available), and annotated details of detected variants (Figure 2B). Furthermore, users can filter variants based on functional types [missense, synonymous, and loss of function (LoF)] or variant types (SNP and Indel) through selection buttons (Figure 2C and D).

**Figure 3:**
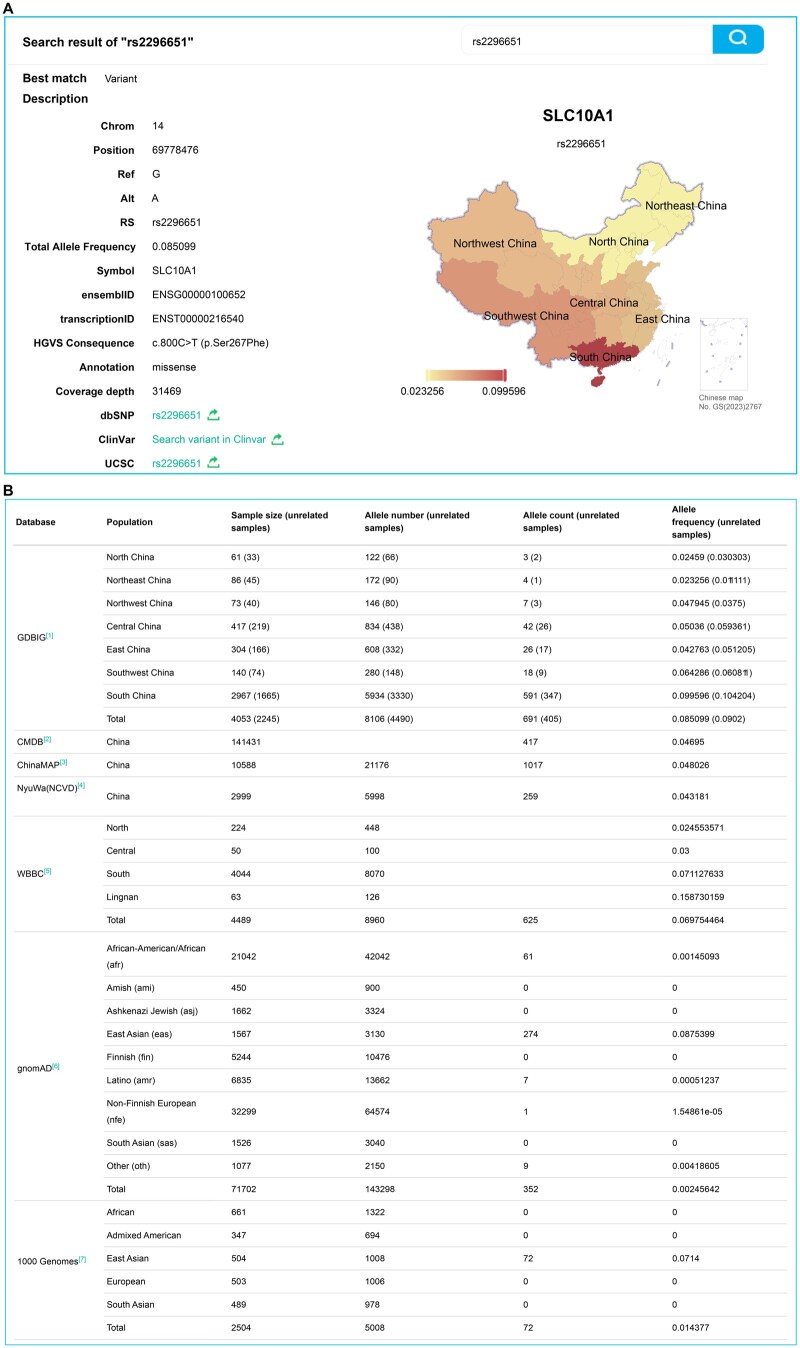
**Example of variant search results in GDBIG** **A**. Search for the rs2296651 variant as an example. The search results display relevant information about the variant, including chromosomal position, allele information, allele frequency, gene ID, and allele frequencies across seven geographical regions of China (North, Northeast, Northwest, East, Central, Southwest, and South). A map of China is provided on the right, where color intensity represents allele frequency, with darker colors indicating higher frequencies. The map of China was obtained from a standard map service [No. GS(2023)2767] approved by the Ministry of National Resources of the People’s Republic of China (http://bzdt.ch.mnr.gov.cn/). **B**. Detailed allele frequency information for the variant in GDBIG, compared to other genomic databases integrated into GDBIG.

By clicking on any search result, users can access more detailed information, including variant descriptions, allele frequency distributions across the seven geographical areas in China, and integrated variant data from global populations available in databases such as CMDB [32], ChinaMAP [30], NyuWa (NCVD) [15], WBBC [30], gnomAD [31], and 1KGP3 [8]. This feature empowers users to investigate variant frequencies from both global and regional perspectives (**Figure 3**), making it particularly valuable for studying rare or localized diseases.

### Genomic API and tools

In addition to the web search page and visualization tools, batch query functionality is critical for addressing specific research objectives. To address this need, we developed a genomic API for GDBIG. This Representational State Transfer (REST) API (https://restfulapi.net/) allows researchers to remotely access variant data via program scripts or command-line operations, offering flexibility and scalability for various computational workflows.

To complement the GDBIG API, we also developed GDBIGtools (https://github.com/BIGCS-Lab/GDBIGtools), a Python-based toolkit designed to streamline genetic variant queries and annotate local variation files in variant call format (VCF). GDBIGtools provides comprehensive data, including overall allele frequency, regional allele frequencies, filtration status, and annotations derived from GDBIG (Figure 1). The toolkit is available on the Python Package Index (PyPI; https://pypi.org/) and can be easily installed using the Python package installer (PIP) by executing the command “pip install GDBIGtools”. Detailed usage instructions are accessible on the API ACCESS page (http://gdbig.bigcs.com.cn/api.html) and the associated GitHub repository. GDBIGtools efficiently processes approximately 1800 variants per minute, with performance contingent on internet connection speed. Query results are output in VCF, ensuring compatibility with standard bioinformatics pipelines and facilitating seamless integration into existing research workflows.

### Genotype imputation server

Genotype imputation is a robust method for accurately inferring individual genotypes at genomic positions not included in genotyping arrays [33]. Utilizing a population-specific reference panel for genotype imputation can significantly enhance the power of GWAS [5,34–37]. Within the BIGCS framework, the inclusion of family members in the study design enables accurate long-range phasing, substantially improving the accuracy of genotype imputation [28]. The current BIGCS reference panel was developed using data from phase I participants of the BIGCS cohort [28], leveraging the unique family relatedness information inherent in the cohort. To construct the reference panel, we selected 2245 unrelated individuals from the phased variant dataset, which contains 43,055,086 high-quality SNPs and 4,184,387 Indels, covering all 22 autosomes and the X chromosome. Notably, this reference panel includes 13.66 million novel variants absent from dbSNP build 154 and 30.88 million variants not previously represented in the 1KGP3 reference panel [8], which has been the primary reference panel for the Chinese population and includes data from 301 unrelated individuals of Chinese ancestry. To evaluate the effectiveness of the BIGCS reference panel in imputing genotypes for individuals of Chinese ancestry, we utilized an independent high-coverage WGS dataset comprising 50 Chinese individuals (40× coverage, 11,174,603 biallelic variants). We simulated a standard imputation process using 930,000 SNP sites from the Affymetrix Genome-Wide Human SNP Array 6.0 and assessed imputation accuracy (*R*^2^) by comparing imputed genotype dosages with true genotypes derived from high-coverage sequencing at sites not included in the array. Across all allele frequency ranges, the BIGCS reference panel consistently demonstrated superior *R*^2^ values and a higher true variant coverage ratio compared to commonly used reference panels for individuals of Chinese ancestry, including the 1KGP3 reference panel (*n* = 2504), the Haplotype Reference Consortium (HRC) panel (*n* = 32,470), the GenomeAsia100K Project (GAsP) reference panel (*n* = 1654), and the multi-ethnic TOPMed reference panel (*n* = 97,256). Specifically, the BIGCS reference panel achieved an average *R*^2^ of 0.715 for low-frequency variants [1% ≤ minor allele frequency (MAF) < 5%] and 0.936 for common variants (MAF ≥ 5%) [28], surpassing the performance of other panels: 1KGP3 [8] (low-frequency variants: 0.687, common variants: 0.922), HRC [38] (low-frequency variants: 0.660, common variants: 0.892), GAsP [39] (low-frequency variants: 0.672, common variants: 0.873), and TOPMed [11] (low-frequency variants: 0.699, common variants: 0.920). For rare variants with MAF ≤ 1%, the BIGCS panel also exhibited the highest average *R*^2^ (*R*^2^ = 0.466) compared to the 1KGP3 (*R*^2^ = 0.291), HRC (*R*^2^ = 0.206), GAsP (*R*^2^ = 0.240), and TOPMed (*R*^2^ = 0.422) panels. Further details about the BIGCS reference panel and its comparative performance are available in our associated published study [28]. For genotype imputation quality control, we recommend sequencing a subset of samples at high depth and empirically evaluating imputation accuracy by comparing true genotypes with imputed genotype dosages.

**Figure 4:**
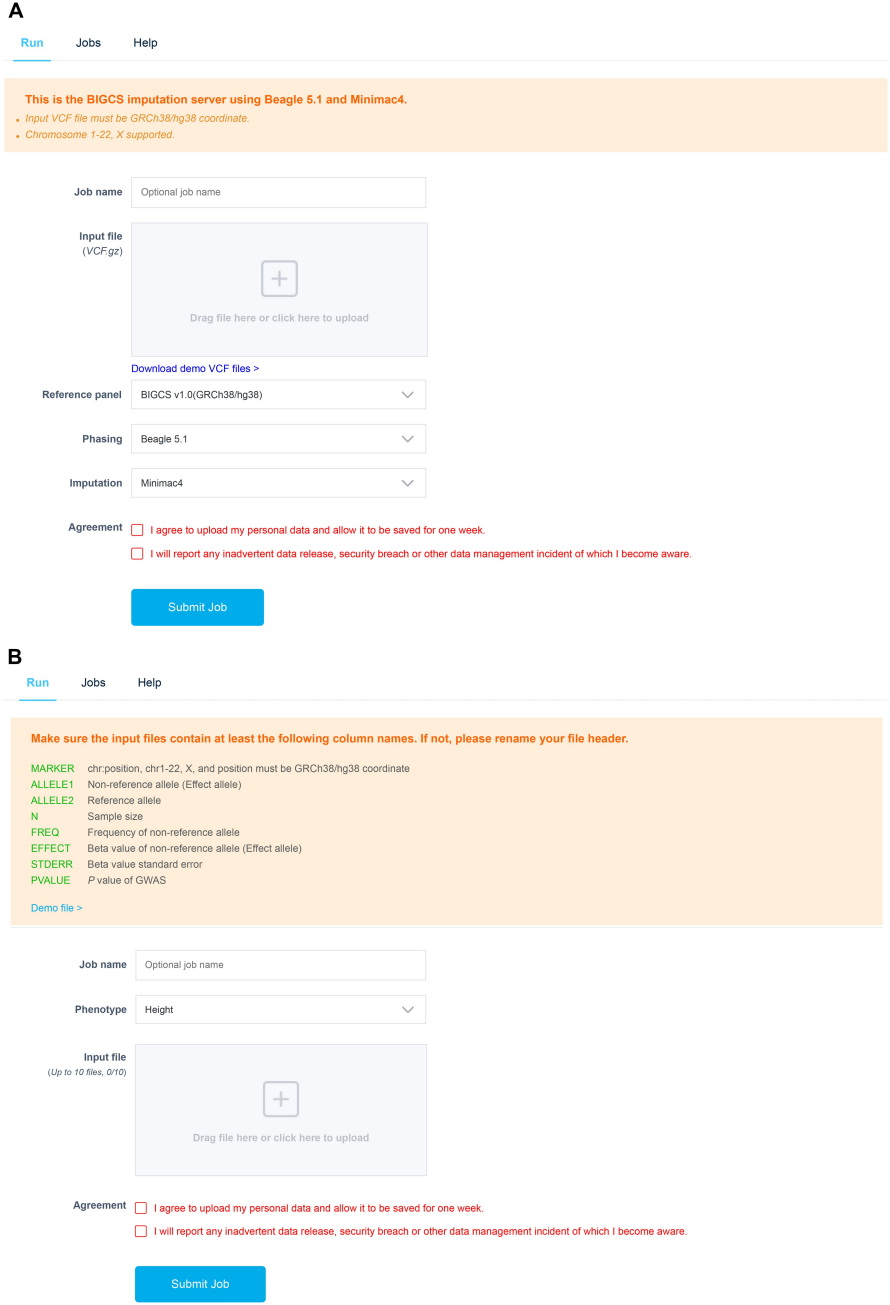
**Web interface for genotype imputation and GWAS meta-analysis** **A**. Web interface for genotype imputation. **B**. GWAS meta-analysis.

Within GDBIG, we have developed a dedicated web interface to facilitate genotype imputation using the BIGCS reference panel. This tool is accessible under the “RESEARCH TOOLS” menu on the GDBIG website (http://gdbig.bigcs.com.cn/imputation_server/jobs.html). The imputation process employs Beagle 5.1 [40] (https://faculty.washington.edu/browning/beagle/b5_1.html) for phasing input VCF files and Minimac4 (https://genome.sph.umich.edu/wiki/Minimac4) for imputation, both with default parameters. The user-friendly workflow involves four steps (**Figure 4**A): (1) Uploading a variant file in VCF format with coordinates conforming to GRCh38/hg38; (2) Selecting the BIGCS reference panel and imputation tools; (3) Submitting the job and awaiting completion; and (4) Downloading the final results. Users will receive email notifications detailing the job status upon task completion. On average, imputing genotypes for a VCF file containing 50 samples and 720 k SNPs takes approximately 1 h (Table S2).

### GWAS meta-analysis interface

GWAS meta-analysis has emerged as a crucial method for improving the statistical power of genetic association studies, particularly in scenarios with limited sample sizes. This approach integrates GWAS summary statistics from multiple studies, enabling analysis across a vast number of genetic variants [41–44]. To support GWAS meta-analysis across a range of phenotypes, including general traits and those specific to pregnancy and fetal growth, the GDBIG platform provides a dedicated portal and workflow powered by the METAL software [45].

In the initial phase of the BIGCS study, we conducted GWAS analyses focusing on anthropometric, gestational, and fetal growth phenotypes. Currently, the GDBIG platform offers publicly accessible GWAS summary statistics for 14 pregnancy-related phenotypes. These include adult anthropometric traits [height, weight, and body mass index (BMI)], lipid metabolism traits during pregnancy [total bile acid (TBA), total cholesterol (TC), high-density lipoprotein (HDL), low-density lipoprotein (LDL), triglycerides (TG)], oral glucose tolerance test (OGTT) levels measured at 24–28 weeks of gestation (0 h, 1 h, 2 h), gestational weight gain (GWG) rate (kg/week), and neonatal birth weight and birth length (**Table 1**). Notably, genetic association signals for phenotypes shared across Chinese pregnancies sequenced through noninvasive prenatal testing (NIPT) [46] achieved 100% replication [28].

**Table 1:** The adult and fetal growth phenotypes included in the GWAS meta-analysis module of GDBIG

Trait	Sample size	Variant number
**Anthropometric trait**		
Height (pre-pregnancy)	2195	7,134,855
Weight (pre-pregnancy)	2165	7,131,603
BMI (pre-pregnancy)	2171	7,132,009
GWG rate	1566	7,110,421
**Biochemical trait (collection in 16–20 weeks of pregnancy)**		
TBA of pregnancy	1695	7,115,228
TC	1535	7,112,498
HDL	1532	7,111,976
LDL	1532	7,111,938
TG	1531	7,111,181
**OGTT (collection in 24–28 weeks of pregnancy)**		
Glucose OGTT 0 h of pregnancy	1684	7,112,787
Glucose OGTT 1 h of pregnancy	1687	7,112,953
Glucose OGTT 2 h of pregnancy	1685	7,112,724
**Fetal growth**		
Birth length	1724	7107,435
Birth weight	1746	7109,094

*Note*: GWAS, genome-wide association study; GDBIG, the Genome Database of BIGCS; BIGCS, the Born in Guangzhou Cohort Study; BMI, body mass index; GWG, gestational weight gain; TBA, total bile acid; TC, total cholesterol; HDL, high-density lipoprotein; LDL, low-density lipoprotein; TG, triglycerides; OGTT, oral glucose tolerance test.

The GWAS meta-analysis workflow on GDBIG is designed to be user-friendly, mirroring the simplicity of the genotype imputation process. It comprises four main steps (Figure 4B): (1) Selecting the phenotype of interest; (2) Uploading one or more GWAS summary statistics files corresponding to the chosen phenotype; (3) Submitting the jobs and awaiting their completion; (4) Downloading the meta-analysis results.

Comprehensive user instructions are available on the GWAS meta-analysis interface page (http://gdbig.bigcs.com.cn/gwas_meta_analysis/status.html). For optimal quality control during meta-analyses, we recommend users evaluate genomic control metrics such as lambda values and quantile–quantile (Q–Q) plots [47], alongside linkage disequilibrium (LD) score regression metrics (intercept and ratio) [48], to monitor genome-wide statistical inflation. Additionally, replication analyses are encouraged to confirm the results of the meta-GWAS.

### Pairwise LD calculation based on the BIGCS variant dataset

**Figure 5:**
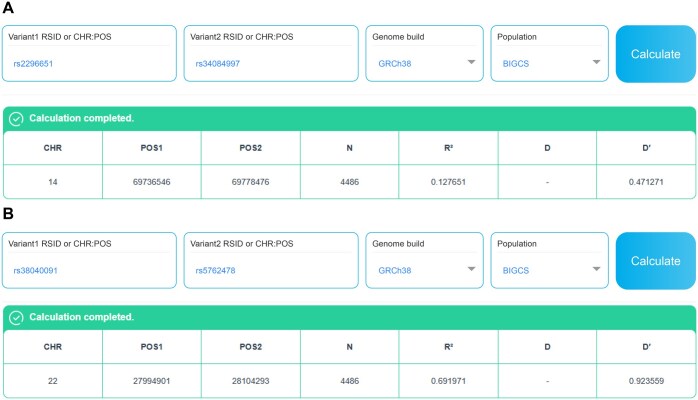
**Examples of pairwise LD calculation** **A**. Pairwise LD calculation between two SNPs: rs2296651 (chr14: 69,778,476, G>A) and rs34084997 (chr14: 69,736,546, C>T). **B**. Pairwise LD calculation between an Indel (rs3840091, chr22: 27,994,901, CCAGA>C) and a SNP (rs5762478, chr22: 28,104,293, T>C).

The GDBIG platform features a module titled “Pairwise LD calculation”, which enables real-time LD analyses utilizing the comprehensive BIGCS variant dataset. This module is accessible via the “RESEARCH TOOLS” menu on the GDBIG website (http://gdbig.bigcs.com.cn/ld/cal.html). Distinct from existing resources, this tool provides LD calculations that include both SNPs and Indels, thereby addressing a critical gap in the field (**Figure 5**). To the best of our knowledge, this is the first online resource specifically designed to perform LD calculations exclusively based on a Chinese population genetic data resource. By incorporating Indels, the tool extends the capabilities of LD analyses, offering researchers a unique and essential resource for studying genetic variation in the Chinese population.

## Discussion and perspectives

The launch of GDBIG represents a significant milestone as the first family-based genetic database and analysis platform linked to the largest birth cohort in China. It addresses the global scarcity of large-scale maternal–infant WGS variation datasets, particularly for the underrepresented Asian population, while providing a comprehensive platform for online genetic data analysis and genomic API utilization. The current release of GDBIG is built upon WGS data from 4053 individuals in phase I of the BIGCS project.

GDBIG offers an extensive array of functionalities, including functional annotations and global/regional allele frequency data for 56.23 million genomic variants. It features a high-quality reference panel with genotype imputation accuracy superior to other Chinese reference panels, a GWAS meta-analysis interface providing genotype–phenotype association results for 14 pregnancy and fetal growth traits, and a dedicated online tool for LD calculations based solely on Chinese population genetic data. Notably, GDBIG facilitates secure data sharing and analysis without requiring raw genetic data disclosure, ensuring privacy while providing researchers with an unparalleled resource for exploring genomic data in the context of a birth cohort. This comprehensive platform paves the way for advancing the understanding of the genetic basis of various traits and diseases, significantly contributing to global genetic and genomic research.

To further enhance GDBIG, we aim to address its current limitations through the following initiatives. First, the current dataset, comprising thousands of participants, restricts allele frequency inference, genotype imputation, and GWAS analyses. As additional genomic data from the BIGCS cohort become available, we plan to expand the database, incorporating new datasets and updating the variant catalog and BIGCS reference panel. We will also integrate human genome variation data from diverse global populations to improve comprehensiveness and representation. Second, the database currently provides GWAS summary statistics for 14 traits. We intend to extend this coverage to include a wider range of maternal pregnancy traits, fetal growth phenotypes, and birth defects. With an expanded sample size, these analyses will gain enhanced statistical power, enabling more robust meta-analyses. Third, the database currently focuses on SNPs and Indels. Recognizing the importance of structural variants (SVs) in genetic diseases and trait etiology [49–51], we plan to incorporate SV data in future releases. This will be supported by high-depth sequencing data from the phase II BIGCS study, further enriching the database’s scope and applicability. Fourth, beyond the existing analyses, we plan to incorporate advanced tools, such as Eagle [52] for haplotype phasing and genotype imputation, and meta-regression of multi-ethnic genetic association (MR-MEGA) [53] for multi-ancestral meta-GWAS analysis. Moreover, we are developing interactive visualization tools for genomic data, which will be available as online applications on the GDBIG platform, which will improve the platform’s analytical capabilities and user experience. Finally, we are committed to refining the functionality and user experience of GDBIGtools. Dedicated user support will be available to assist researchers in navigating the platform effectively and maximizing its utility. Through these efforts, we aim to ensure that GDBIG remains a state-of-the-art resource for genetic research, supporting investigations into intergenerational and population genetics while advancing scientific discovery on a global scale.

## Ethical statement

The study was approved by the Ethics Committee of GWCMC, China (Approval No. 2017102302). All participants provided written informed consent for the use of their data for research purposes.

## Code availability

The ilus software (utilized for variant calling in BIGCS data) and GDBIGtools are available in public GitHub repositories at https://github.com/ShujiaHuang/ilus and https://github.com/BIGCS-Lab/GDBIGtools, respectively. Both ilus and GDBIGtools have also been submitted to BioCode at the National Genomics Data Center (NGDC) [54], China National Center for Bioinformation (CNCB) (BioCode: BT007705 and BT007586, respectively), which are publicly accessible at https://ngdc.cncb.ac.cn/biocode/tool/BT007705 and https://ngdc.cncb.ac.cn/biocode/tool/BT007586.

## Supplementary Material

qzaf045_Supplementary_Data

## Data Availability

The raw sequencing data underlying GDBIG have been deposited in the Genome Sequence Archive for Human [55] at the NGDC [54], CNCB (GSA-Human: HRA002496), and are publicly accessible at https://ngdc.cncb.ac.cn/gsa-human/. The data can be accessed through applications following guidelines provided in the GSA-Human documentation (https://ngdc.cncb.ac.cn/gsa-human/document). All GWAS summary statistics data hosted on the GDBIG platform have been deposited in the Genome Variation Map [56] at the NGDC [54], CNCB (GVM: GVP000003), and are publicly accessible at https://ngdc.cncb.ac.cn/gvm/getProjectDetail?project=GVP000003. Users interested in accessing these datasets may contact the corresponding author to request permission. GDBIG has been submitted to Database Commons [57] at the NGDC, CNCB, which is publicly accessible at https://ngdc.cncb.ac.cn/databasecommons/database/id/9713. Researchers can utilize the platform via an interactive API, available at http://gdbig.bigcs.com.cn/api.html, where tutorials are provided to guide users in navigating the system. Variant dataset and the GWAS summary statistics for the 14 traits can also be accessed through the GDBIG server (http://gdbig.bigcs.com.cn/), with approval from the Human Genetic Resources Administration of China (HGRAC) (ID: 2022BAT1051). Researchers interested in collaborating with the BIGCS group are encouraged to contact Xiu Qiu (xiu.qiu@bigcs.org) or the data support team at data.bigcs@bigcs.org.

## CRediT author statement

**Shujia Huang:** Conceptualization, Investigation, Methodology, Project administration, Formal analysis, Software, Visualization, Validation, Writing – original draft, Writing – review & editing, Funding acquisition. **Chengrui Wang:** Investigation, Data curation, Formal analysis, Software, Visualization, Writing. **Mingxi Huang:** Investigation, Data curation, Formal analysis, Visualization, Validation. **Jinhua Lu:** Platform, Validation. **Jian-Rong He:** Validation, Writing – review & editing. **Shanshan Lin:** Validation, Writing – review & editing. **Siyang Liu:** Validation, Writing – review & editing. **Huimin Xia:** Funding acquisition, Resources. **Xiu Qiu:** Conceptualization, Supervision, Writing – review & editing, Funding acquisition, Resources, Validation, Project administration. All authors have read and approved the final manuscript.
