# Supplementary material for: GDBIG: A Pioneering Birth Cohort Genomic Platform Facilitating Intergenerational Genetic Research
Source: Genomics Proteomics Bioinformatics. 2025 May 15;23(5):qzaf045. doi: 10.1093/gpbjnl/qzaf045 (PMC12944825; doi:10.1093/gpbjnl/qzaf045)
Supplement: qzaf045_Supplementary_Data [file qzaf045_supplementary_data.zip › Supplementary material captions.docx]

**Supplementary material**

**Figure S1 Demographic characteristics of the 4053 BIGCS phase I samples in GDBIG**

**A.** Age distribution of parental participants. The Y-axis indicates the sample size, while each color bar along the X-axis represents a specific age. Males are shown in blue and females in pink. **B.** Pie chart showing the parental sex distribution of the participants, with males represented in blue and females in pink. **C.** Pie chart showing the children’s sex distribution of the participants, with males represented in blue and females in pink. **D.** Ethnic distribution of the participants. The Y-axis represents sample size on a log scale, and each color bar on the X-axis represents an ethnic group, with the sample size indicated in parentheses. **E.** Dialect distribution of the participants. The Y-axis represents sample size on a log scale, and each color bar on the X-axis represents a dialect group, with sample size indicated in parentheses.

**Table S1 Geographical distribution of the participants**

**Table S2** **Time elapsed for ten replicated genotype imputation experiments for a VCF file containing 50 samples and 720 k SNP genotyping array data using the BIGCS reference panel in GDBIG**
