# Supplementary figures and images for: GDBIG: A Pioneering Birth Cohort Genomic Platform Facilitating Intergenerational Genetic Research
Source: Genomics Proteomics Bioinformatics. 2025 May 15;23(5):qzaf045. doi: 10.1093/gpbjnl/qzaf045 (PMC12944825; doi:10.1093/gpbjnl/qzaf045)

A

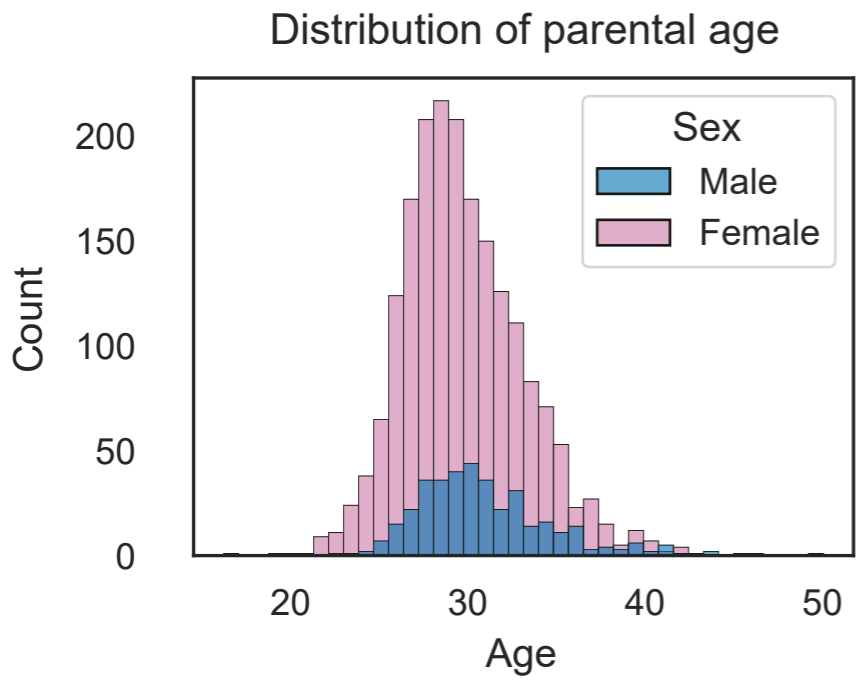

B

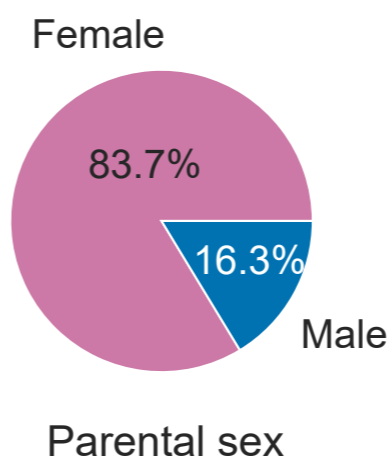

C

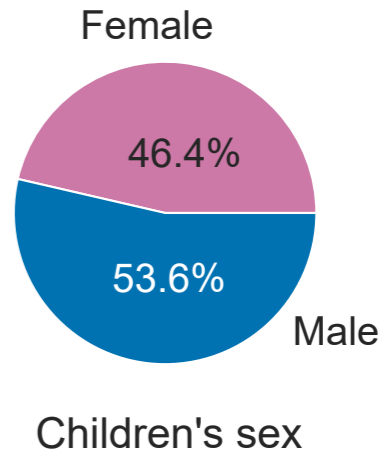

D

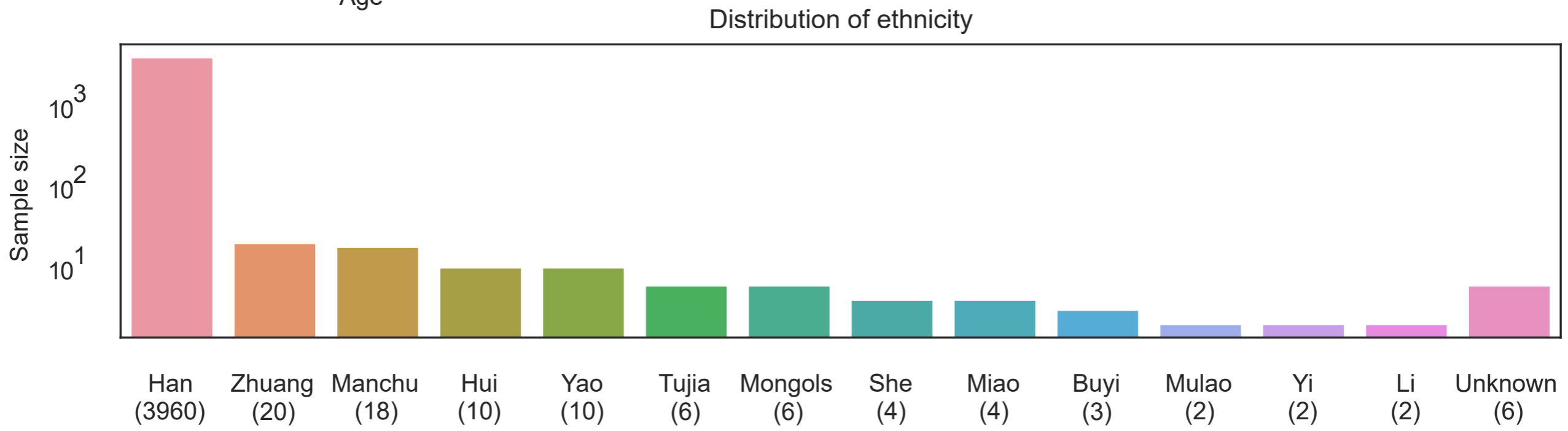

E

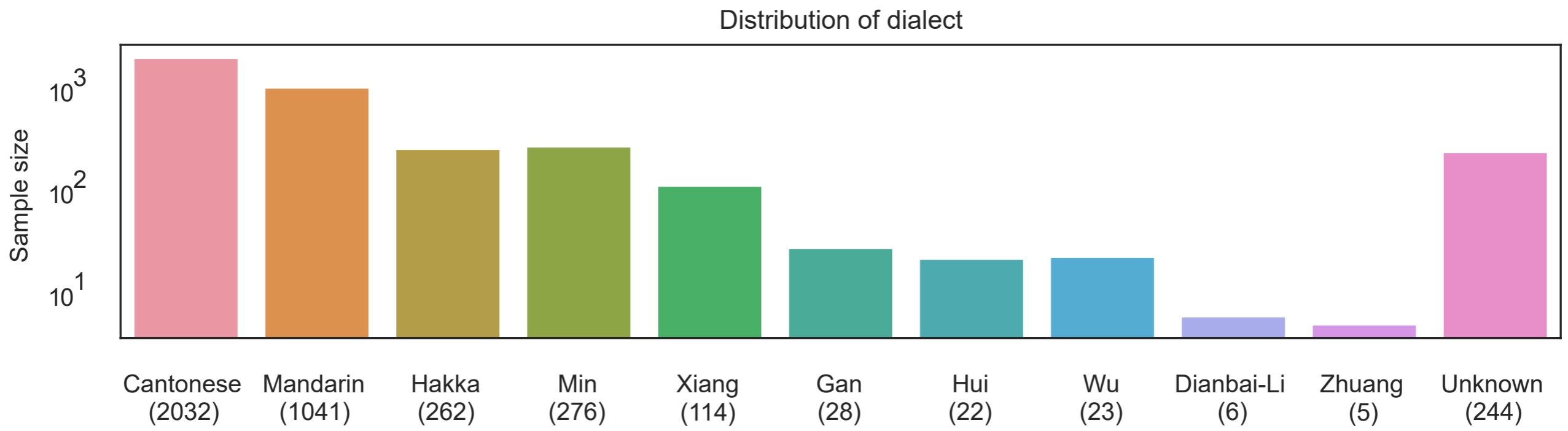

Supplement: qzaf045_Supplementary_Data [file qzaf045_supplementary_data.zip › Figure S1.pdf]
